# Neuroanatomy of the crocodylian *Tomistoma dowsoni* from the Miocene of North Africa provides insights into the evolutionary history of gavialoids

**DOI:** 10.1111/joa.13846

**Published:** 2023-03-16

**Authors:** Paul M. J. Burke, Philip D. Mannion

**Affiliations:** ^1^ Department of Earth Sciences University College London London UK

**Keywords:** computed tomography, crocodylian, ecomorphology, gharial, morphometrics neuroanatomy, Tomistominae

## Abstract

The interrelationships of the extant crocodylians *Gavialis gangeticus* and *Tomistoma schlegelii* have been historically disputed. Whereas molecular analyses indicate a sister taxon relationship between these two gavialoid species, morphological datasets typically place *Gavialis* as the outgroup to all other extant crocodylians. Recent morphological‐based phylogenetic analyses have begun to resolve this discrepancy, recovering *Gavialis* as the closest living relative of *Tomistoma*; however, several stratigraphically early fossil taxa are recovered as closer to *Gavialis* than *Tomistoma*, resulting in anomalously early divergence timings. As such, additional morphological data might be required to resolve these remaining discrepancies. ‘*Tomistoma*’ *dowsoni* is an extinct species of gavialoid from the Miocene of North Africa. Utilising CT scans of a near‐complete, referred skull, we reconstruct the neuroanatomy and neurosensory apparatus of ‘*Tomistoma*’ *dowsoni*. Based on qualitative and quantitative morphometric comparisons with other crocodyliforms, the neuroanatomy of ‘*Tomistoma*’ *dowsoni* is characterised by an intermediate morphology between the two extant gavialoids, more closely resembling *Gavialis*. This mirrors the results of recent studies based on the external anatomy of these three species and other fossil gavialoids. Several neuroanatomical features of these species appear to reflect ecological and/or phylogenetic signals. For example, the ‘simple’ morphology of their neurosensory apparatus is broadly similar to that of other long and narrow‐snouted (longirostrine), aquatic crocodyliforms. A dorsoventrally short, anteroposteriorly long endosseous labyrinth is also associated with longirostry. These features indicate that snout and skull morphology, which are themselves partly constrained by ecology, exert an influence on neuroanatomical morphology, as has also been recognised in birds and turtles. Conversely, the presence of a pterygoid bulla in *Gavialis* and several extinct gavialoids, and its absence in *Tomistoma schlegelii*, could be interpreted as a phylogenetic signal of crocodylians more closely related to *Gaviali*s than to *Tomistoma*. Evaluation of additional fossil gavialoids will be needed to further test whether these and other neuroanatomical features primarily reflect a phylogenetic or ecological signal. By incorporating such previously inaccessible information of extinct and extant gavialoids into phylogenetic and macroecological studies, we can potentially further constrain the clade's interrelationships, as well as evaluate the timing and ecological association of the evolution of these neuroanatomical features. Finally, our study supports recent phylogenetic analyses that place ‘*Tomistoma*’ *dowsoni* as being phylogenetically closer to *Gavialis gangeticus* than to *Tomistoma schlegelii*, indicating the necessity of a taxonomic revision of this fossil species.

## Graphical abstract

We compared the neuroanatomy of *Tomistoma dowsoni*, a fossil gavialoid crocodylian from the Miocene of North Africa, to the two extant gavials, *Gavialis gangeticus* and *Tomistoma schlegelii*. *Tomistoma dowsoni* displays an intermediate morphology between the two extant gavials, with a closer resemblance to *Gavialis*. Our study identifies several neuroanatomical features with an apparent ecological signal and reveals new insights into the evolutionary history of gavialoids.

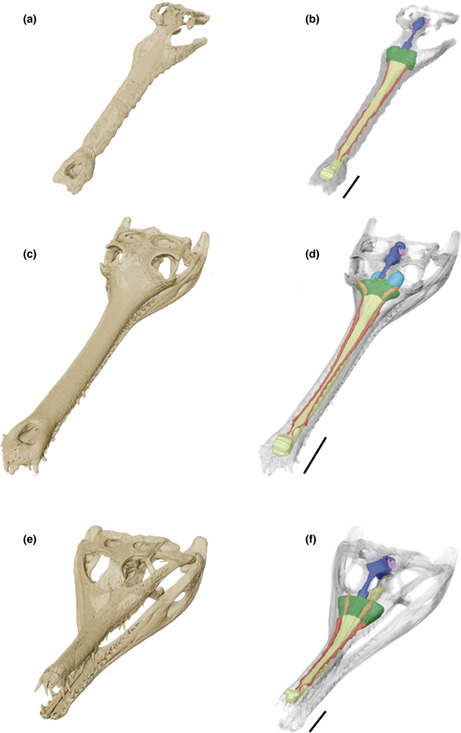

## 1 INTRODUCTION

Crocodylia is a clade of semi‐aquatic, ambush predators that inhabit both freshwater and estuarine environments (Grigg & Kirshner, 2015). They are broadly restricted to the subtropical latitudinal belt, with over 25 extant species currently recognised, consisting of alligators, caimans, crocodiles and gavials (Grigg & Kirshner, 2015). Crocodylian interrelationships have been debated for decades, most notably due to conflicting results from phylogenetic analyses based on molecular (Densmore & Owen, 1989) versus morphological (Brochu, 1997) data. The most notable discrepancy pertains to the position of the extant species *Gavialis gangeticus*: whereas molecular data recover it as the closest living relative of *Tomistoma schlegelii*, forming the clade Gavialidae, morphological data have typically placed it as the sister taxon to all other extant crocodylians (Brochu, 1997). For the first time based solely on morphological data, Rio and Mannion (2021) robustly recovered *Gavialis gangeticus* as the closest living relative of *Tomistoma schlegelii* (see also Ristevski et al., 2022). Additionally, their analyses recovered more widespread similarities between fossil taxa traditionally referred to Tomistominae and Gavialoidea (see also Iijima et al., 2022; Iijima & Kobayashi, 2019). Despite this, a temporal incongruence is still evident in Gavialoidea, with several stratigraphically early fossil taxa recovered as closer to *Gavialis* than *Tomistoma*, resulting in inferred divergence timings for Gavialidae that greatly predate those estimated from molecular data (Rio & Mannion, 2021). Although the incorporation of further taxa (e.g. Iijima et al., 2022), as well as methodological approaches such as total evidence analyses (Darlim et al., 2022; Lee & Yates, 2018), might help resolve this discrepancy, one potential additional resource might emanate from the internal anatomy of specimens (Gold et al., 2014), utilising previously inaccessible data increasingly made available through computed tomography.

Evaluation of neuroanatomy via computed tomography is a relatively new approach, with the first applications to crocodylians and their extinct relatives by Rowe et al. (1999) and Tykoski et al. (2002), who presented data on *Alligator mississippiensis* and the Early Jurassic goniopholid neosuchian *Calsoyasuchus valliceps* respectively. Since then, neuroanatomical reconstructions of the group have included studies of extant species (e.g. Dufeau & Witmer, 2015; Gold et al., 2014; Kuzmin et al., 2021; Lessner & Holliday, 2022; Witmer et al., 2008; Witmer & Ridgely, 2008), as well as an increasing number of extinct taxa, including early diverging crocodylomorphs (Leardi et al., 2020; Melstrom et al., 2022; Ruebenstahl et al., 2022), thalattosuchians (e.g. Herrera et al., 2018; Pierce et al., 2017; Schwab et al., 2021; Wilberg et al., 2022), notosuchians (e.g. Dumont Jr et al., 2022; Kley et al., 2010; Pochat‐Cottilloux et al., 2021; Sereno & Larsson, 2009; Sertich & O'connor, 2014) and eusuchian taxa outside of the crocodylian radiation (Blanco et al., 2015; Holliday & Gardner, 2012; Puértolas‐Pascual et al., 2022; Serrano‐Martínez et al., 2019, 2021). Within Crocodylia, there are surprisingly few published neuroanatomical reconstructions of extinct species, limited to the alligatoroid *Diplocynodon tormis* (Serrano‐Martínez et al., 2019), the mekosuchines *Paludirex vincenti* (Ristevski et al., 2020) and *Trilophosuchus rackhami* (Ristevski, 2022), the caimanine *Mourasuchus arendsi* (Bona et al., 2013), and the gavialoids *Gryposuchus neogaeus* (Bona et al., 2015) and *Gunggamarandu maunala* (Ristevski et al., 2021). Although many lineages remain unstudied, these analyses are beginning to reveal evolutionary transitions in crocodylomorph neuroanatomy, including features that appear to be unique to individual clades (e.g. Barrios et al., 2023; Ruebenstahl et al., 2022; Schwab et al., 2020; Serrano‐Martínez et al., 2021). However, they also document morphological similarities between distantly related taxa (e.g. *Gavialis* and Thalattosuchia), mirroring patterns in external anatomy (e.g. Ballell et al., 2019; Brochu, 2001; Felice et al., 2021; Groh et al., 2020; Jouve, 2009).

Here, we present a reconstruction of the neuroanatomy and neurosensory apparatus of the Miocene North African fossil crocodylian species ‘*Tomistoma*’ *dowsoni*. In recent phylogenetic analyses, ‘*Tomistoma*’ *dowsoni* has been recovered as a gavialoid, more closely related to *Gavialis gangeticus* than to *Tomistoma schlegelii* (Groh et al., 2020; Rio & Mannion, 2021). Given its ‘intermediate’ position within Gavialoidea (sensu Iijima et al., 2022), the neuroanatomy of both extant gavialoids is also reconstructed for comparative purposes. Additionally, we quantitatively evaluate morphological variation in crocodyliform neuroanatomy, especially that of crocodylians and their close relatives, as well as test how this corresponds to the environment they inhabit.

*Institutional abbreviations*. AMNH; American Museum of Natural History, New York City, New York, USA; BSPG, Bavarian State Collection for Palaeontology and Geology, Munich, Germany; BP, Evolutionary Studies (formerly Bernard Price) Institute, Johannesburg, South Africa; FLMNH, Florida Museum of Natural History, Gainesville, Florida, USA; IVPP, Institute of Vertebrate Paleontology and Paleoanthropology, Beijing, China; MLP, Museo de La Plata, Buenos Aires, Argentina; NMB, National Museum of the Bahamas, Nassau, Bahamas; NHMUK, Natural History Museum, London, UK; OUVC, Ohio University Vertebrate Collection, Athens, Ohio, USA; QMF, Queensland Museum, Brisbane, Queensland, Australia; TMM, Texas Memorial Museum, Austin, Texas, USA; UMZC, University Museum of Zoology, Cambridge, UK; USNM, Smithsonian Institution National Museum of Natural History, Washington D.C., USA.

## 2 MATERIALS AND METHODS

### 2.1 Specimens and CT scan reconstructions

The neuroanatomy of ‘*Tomistoma*’ *dowsoni* was interpreted from NHMUK PV R 4769. This is a referred specimen purchased by the NHMUK in 1920 from Lady Moon, and it was collected from near the Siwa Oasis, in the Western Desert of Egypt (Hamilton, 1973). Its precise stratigraphic provenance is uncertain, but it is likely to be from the lower Miocene Moghra (=Moghara) Formation (Hamilton, 1973). NHMUK PV R 4769 has been used as the basis for the ‘*Tomistoma*’ *dowsoni* operational taxonomic unit in recent phylogenetic analyses (Groh et al., 2020; Rio & Mannion, 2021), in which it has been recovered as more closely related to *Gavialis gangeticus* than to *Tomistoma schlegelii*. It is represented by a near‐complete and well‐preserved skull, missing the quadratojugals, pterygoids and ectopterygoids. The specimen is supported by a longitudinal metal rod which affects the reconstruction of some neuroanatomical features. Similarly, an incomplete left squamosal and postorbital also impacted reconstruction of the paratympanic region.

NHMUK PV R 4769 was characterised at the NHMUK with X‐ray micro‐computed tomography using a Nikon Metrology XTH 225 ST system (Nikon Metrology, Leuven, Belgium). Acquisition of the full skull was implemented in five parts, with a voltage of 215 kV and a current of 698 μA, resulting in a reconstructed isotropic voxel size of 75.999 μm^3^, and 4476 projections with an average of four frames, with an exposure time of 0.708 seconds per frame. Three acquisitions were carried out for the posterior portion of the skull, followed by the skull being turned upside‐down and the last two acquisitions captured the anterior portion of the skull. Datasets were merged into a single volume using Avizo v. 9.7 (FEI Visualization Science Group; https://www.thermofisher.com ), using the protocol described in (Butler et al., 2022). The neuroanatomy of NHMUK PV R 4769 was subsequently segmented in Avizo v. 9.7, smoothed in Blender (Stichting Blender Foundation, Amsterdam) and rendered in Inkscape (Inkscape Project, 2020).

The neuroanatomy of the extant gavialoids *Gavialis gangeticus* (FLMNH UF 118998) and *Tomistoma schlegelii* (TMM M6342) was reconstructed based on data available in MorphoSource (https://www.morphosource.org/). As the paratympanic region is not preserved in NHMUK PV R 4769, these features were not segmented in *Gavialis gangeticus* and *Tomistoma schlegelii*. Both specimens used are adults, for accurate comparison to NHMUK PV R 4769, as brain volume varies throughout ontogeny (Jirak & Janacek, 2017; Watanabe et al., 2019). These two extant taxa, as well as published neuroanatomical reconstructions of the extinct gavialoid *Gryposuchus neogaeus* from the Miocene of Argentina (Bona et al., 2015), the non‐crocodylian allodaposuchid eusuchian *Agaresuchus fontisensis* from the Late Cretaceous of Spain (Serrano‐Martínez et al., 2021), and the thalattosuchian *Pelagosaurus typus* from the Early Jurassic of the UK (Pierce et al., 2017), were used as a comparative framework during segmentation.

### 2.2 Reptile encephalisation quotient

The reptile encephalisation quotient (REQ) was developed by Hurlburt (1996) from the encephalisation Quotient of Jerison (1973), based on extant reptile species. The REQ is a commonly used metric to measure relative brain size of extinct species (Paulina‐Carabajal & Currie, 2017), and has been previously applied to eusuchian neuroanatomy to infer cognitive capabilities (Serrano‐Martínez et al., 2021). Measuring REQ requires an estimation of body and brain mass. Body mass was calculated for NHMUK PV R 4769 using the regression equation Ln (Total Length of Skull) = 0.32Ln (Body Mass) + 2.05 (Platt et al., 2009), which was subsequently rearranged to interpret body mass: Body mass = (Total Length of Skull x e^−2.05^)^1/0.32^. Brain mass was estimated using the endocast volume, applying a density of 1 g/cm^3^ (Franzosa, 2004). As the endocast volume would not necessarily be the same as the brain volume, given that the endocast represents the brain and its associated tissues (Hopson & Gans, 1979; Jirak & Janacek, 2017; Watanabe et al., 2019), the relative brain volume was estimated using a linear regression derived by Serrano‐Martínez et al. (2021) using data published by Jirak and Janacek (2017) and Watanabe et al. (2019). The REQ was subsequently calculated using the equation, REQ = MBr/(0.0155 × MBd^0.553^), where MBr is the mass of the brain and MBd is the body mass (Hurlburt et al., 2013; Paulina‐Carabajal & Currie, 2017).

### 2.3 Olfactory capability and visual acuity calculations

The olfactory capabilities of ‘*Tomistoma*’ *dowsoni* were calculated using the methodology of Zelenitsky et al. (2011). Olfaction acuity is dependent on the size of mitral cells, as well as odour receptors, which can be estimated from the relative size of the olfactory bulb (Lautenschlager et al., 2012; Serrano‐Martínez et al., 2019; Zelenitsky et al., 2009). The greatest diameter of the olfactory bulb of each of NHMUK PV R 4769, *Gavialis gangeticus*, and *Tomistoma schlegelii* was compared to the greatest diameter of their respective cerebrum hemispheres, which was subsequently normalised via a log transformation (Serrano‐Martínez et al., 2021).

Visual acuity is usually estimated from the size of the eyeball, which can be inferred from the sclerotic ring (Lautenschlager et al., 2012). As eusuchians lack sclerotic rings, Serrano‐Martínez et al. (2021) estimated the relative size of the optic region using the optic lobes, which can be inferred from the rhombencephalon region of the endocast (Jirak & Janacek, 2017). The relative volume of the optic region was calculated with the Arithmetic function in Avizo v. 9.7, by comparing the volume of the optic lobe to the volume of the whole endocast.

### 2.4 Morphometric data

Morphometric data were collated from the endocasts and endosseous labyrinths of NHMUK PV R 4769, *Gavialis gangeticus* and *Tomistoma schlegelii*, using the ‘Measurement’ tool in Avizo v. 9.7 (Table 1). Selected dimensions followed Pierce et al. (2017). Our dataset was augmented by measurements from specimens of taxa presented in the published literature (Erb & Turner, 2021; Pierce et al., 2017; Ristevski, 2022), namely: the thalattosuchians *Pelagosaurus typus* and *Plagiophthalmosuchus* cf. *gracilirostris*; the dyrosaurid *Rhabdognathus aslerensis*; the mekosuchine *Trilophosuchus rackhami*; and several extant taxa, comprising *Alligator mississippiensis*, *Caiman crocodilus*, *Crocodylus johnstoni*, *Crocodylus niloticus* and an additional specimen of *Gavialis gangeticus* (UMZC R5792). We also collected morphometric data by measuring published digital endocasts and endosseous labyrinths of a third specimen of *Gavialis gangeticus* (MLP 602; Bona et al., 2015), *Gyprosuchus neogaeus* (Bona et al., 2015), the Miocene South American caimanine *Mourasuchus grendsi* (Bona et al., 2013), and the allodaposuchids *Agaresuchus fontisensis* (Serrano‐Martínez et al., 2021) and *Arenysuchus gascabadiolorum* (Puértolas‐Pascual et al., 2022). Following Pierce et al. (2017), we converted the raw morphometric data into ratios, in order to interpret the relative proportions of the olfactory tract, cerebrum, pituitary fossa and the endosseous labyrinth (Table 2).

**TABLE 1:** Measurements of the endocasts and labyrinths of crocodyliform taxa.

Measurements (mm)	*Tomistoma dowsoni*	*Tomistoma schlegelii*	*Gavialis gangeticus*	*Gavialis gangeticus*	*Gavialis gangeticus*	*Agaresuchus fontisensis*	*Alligator mississippiensis*	*Arenysuchus gascabadiolorum*
	This study	This study	This study	Pierce et al. (2017)	Bona et al. (2015)	Serrano‐Martínez et al. (2021)	Witmer and Ridgely (2008)	Puértolas‐Pascual et al. (2022)
Skull width at cerebrum	109	68	135	168	?	106	73	70
Cephalic flexure angle	143	134	155	150	144	149	135	154
Pontine flexure angle	149	134	119	154	148	149	145	152
Endocast length	146	97	120	146	134	107	98	54
Olfactory tract length	66	47	49	55	52	43	48	20
Cerebrum width	30	27	28	32	31	20	21	18
Pituitary width	9	5	7	6	8	7	5	4
Pituitary height	9	8	9	9	10	11	8	5
Pituitary length	17	14	16	11	10	6	10	7
Labyrinth height	19	18	19	21	14	?	18	?
Labyrinth width	15	17	17	21	17	?	14	?
Cochlear duct length	11	10	10	9	7	?	8	?
Anterior semi‐circular canal area	21	17	20	36	26	?	35	?
Posterior semi‐circular canal area	10	5	6	15	10	?	12	?
Lateral semi‐circular canal area	?	4	10	22	14	?	13	?

Measurements (mm)	*Caiman crocodilus*	*Crocodylus johnstoni*	*Crocodylus niloticus*	*Gryposuchus neogaeus*	*Pelagosaurus typus*	*Rhabdognathus aslerensis*	*Plagiophthalmosuchus* cf. *gracilirostris*	*Trilophosuchus rackhami*
	Jirak and Janacek (2017)	Witmer et al. (2008)	Jirak and Janacek (2017)	Bona et al. (2015)	Pierce et al. (2017)	Erb and Turner (2021)	Brusatte et al. (2016)	Ristevski (2022)
Skull width at cerebrum	?	?	?	243	52	89	?	37
Cephalic flexure angle	138	145	152	?	160	158	175	136
Pontine flexure angle	162	153	160	?	160	152	170	142
Endocast length	71	103	113	?	57	171	?	56
Olfactory tract length	34	46	58	?	21	104	?	22
Cerebrum width	21	29	33	42	15	26	28	17
Pituitary width	?	5	?	16	6	5	14	4
Pituitary height	?	8	?	13	7	10	12	2
Pituitary length	?	11	?	?	10	14	17	6
Labyrinth height	?	13	?	23	14	25	26	13
Labyrinth width	?	14	?	24	11	26	26	10
Cochlear duct length	?	6	?	8	8	12	13	6.5
Anterior semi‐circular canal area	?	18	?	?	9	19	38	8
Posterior semi‐circular canal area	?	5	?	?	6	4	19	4
Lateral semi‐circular canal area	?	8	?	18	4	8	14	2

**TABLE 2:** Ratios of endocast and labyrinth proportions of crocodyliform taxa.

Measurements (mm)	*Tomistoma dowsoni*	*Tomistoma schlegelii*	*Gavialis gangeticus*	*Gavialis gangeticus*	*Gavialis gangeticus*	*Agaresuchus fontisensis*	*Alligator mississippiensis*	*Arenysuchus gascabadiolorum*
	This study	This study	This study	Pierce et al. (2017)	Bona et al. (2015)	Serrano‐Martínez et al. (2021)	Witmer and Ridgely (2008)	Puértolas‐Pascual et al. (2022)
Cerebrum width: Skull width	0.27	0.39	0.21	0.19	?	0.18	0.28	0.26
Cerebrum width: Endocast length	0.21	0.28	0.23	0.22	0.23	0.18	0.21	0.33
Olfactory tract length: Endocast length	0.45	0.48	0.41	0.38	0.38	0.40	0.48	0.37
Pituitary width: Pituitary height	1.00	0.63	0.78	0.67	0.80	0.63	0.63	0.80
Pituitary width: Pituitary length	0.53	0.36	0.44	0.55	0.80	1.16	0.50	0.57
Pituitary length (Endocast length–Olfactory tract length)	0.21	0.28	0.23	0.12	0.12	0.09	0.20	0.21
Labyrinth width: Labyrinth height	0.79	0.94	0.89	1.00	1.21	?	0.78	?
Cochlear duct length: Labyrinth height	0.58	0.56	0.53	0.43	0.50	?	0.44	?
Anterior semi‐circular canal area: Posterior semi‐circular canal area	2.10	3.40	3.33	2.40	2.60	?	2.91	?
Anterior semi‐circular canal area: Lateral semi‐circular canal area	?	4.25	2.00	1.64	1.86	?	2.69	?
Posterior semi‐circular canal area: Lateral semi‐circular canal area	?	1.25	0.60	0.68	0.71	?	0.92	?

Measurements (mm)	*Caiman crocodilus*	*Crocodylus johnstoni*	*Gryposuchus neogaeus*	*Pelagosaurus typus*	*Rhabdognathus aslerensis*	*Plagiophthalmosuchus* cf. *gracilirostris*	*Trilophosuchus rackhami*
	Jirak and Janacek (2017)	Witmer et al. (2008)	Bona et al. (2015)	Pierce et al. (2017)	Erb and Turner (2021)	Brusatte et al. (2016)	Ristevski (2022)
Cerebrum width: Skull width	?	?	0.17	0.28	0.29	?	0.45
Cerebrum width: Endocast length	0.29	0.28	?	0.26	0.15	?	0.30
Olfactory tract length: Endocast length	0.48	0.45	?	0.37	0.61	?	0.39
Pituitary width: Pituitary height	?	0.63	1.23	0.86	0.50	1.17	2.00
Pituitary width: Pituitary length	?	0.45	?	0.60	0.36	0.82	0.66
Pituitary length (Endocast length–Olfactory tract length)	?	0.19	?	0.38	0.21	?	0.18
Labyrinth width: Labyrinth height	?	1.08	?	0.79	1.04	1.00	1.30
Cochlear duct length: Labyrinth height	?	0.46	?	0.57	0.48	0.50	0.50
Anterior semi‐circular canal area: Posterior semi‐circular canal area	?	3.60	?	1.50	4.75	2.00	2.00
Anterior semi‐circular canal area: Lateral semi‐circular canal area	?	2.25	?	2.25	2.38	2.71	4.00
Posterior semi‐circular canal area: Lateral semi‐circular canal area	?	0.63	?	1.50	0.50	1.36	2.00

Variation in the shape of the endosseous labyrinth was measured using 82 curved, semi‐landmarks plotted around the inner ear, as well as around each of the semi‐circular canals. We added ‘*Tomistoma*’ *dowsoni* to the dataset collated by Ristevski (2022), which consists of the morphologies of endosseous labyrinths across 20 crocodylomorphs, including terrestrial, semi‐aquatic and pelagic species. Due to most species included in this study having a semi‐aquatic ecology, species were also classified based on their skull morphology. Adapting the classification system of Busbey (1995), the ratio of the rostrum length compared to the skull length is less than 0.55 in short‐snouted/‘brevirostrine’ taxa, 0.55 to 0.7 in ‘mesorostrine’ taxa, and greater than 0.7 in long‐snouted/‘longirostrine’ taxa. Skull width was also measured at the premaxilla and at the orbits, in order to determine the snout thickness (see Table 3). In this study, we use the term ‘longirostrine’ to describe crocodyliform skulls that have a long and narrow snout compared to the skull table and ‘brevirostrine’ to describe skulls that have a snout with a relatively similar width to the skull table (Table 3).

**TABLE 3:** Table of specimens of Crocodylomorpha used in the principal component analysis (PCA) measuring variation in the shape of the endosseous labyrinth.

Species	Specimen number	Reference	Ecology	Group	Skull‐rostrum length ratio	Skull width ratio	Skull morphology
*Alligator mississippiensis*	USNM 211233	(Dufeau & Witmer, 2015)	Semi‐aquatic	Alligatoridae	0.44	0.62	Brevirostrine
*Caiman crocodilus*	FMNH 73711	(Brusatte et al., 2016)	Semi‐aquatic	Crocodylidae	0.64	0.31	Mesorostrine
*Cricosaurus araucanensis*	MLP 72‐IV‐7‐1	(see Ristevski, 2022)	Pelagic	Thalattosuchia	0.66	0.17	Longirostrine
*Crocodylus johnstoni*	OUVC 10425	(Brusatte et al., 2016)	Semi‐aquatic	Crocodylidae	0.63	0.38	Mesorostrine
*Crocodylus rhombifer*	NMB AB50.0171	(see Ristevski, 2022)	Semi‐aquatic	Crocodylidae	0.65	0.45	Mesorostrine
*Gavialis gangeticus*	FLMNH UF 118998	This study	Semi‐aquatic	Gavialoidea	0.78	0.29	Longirostrine
*Gavialis gangeticus*	UMZC R 5792	(Pierce et al., 2017)	Semi‐aquatic	Gavialoidea	0.78	0.29	Longirostrine
*Gryposuchus neogaeus*	MLP 68‐IX‐V‐1	(Bona et al., 2015)	Semi‐aquatic	Gavialoidea	0.78	0.28	Longirostrine
*Gunggamarandu maunala*	QMF 548	(see Ristevski, 2022)	Semi‐aquatic	Tomistominae	0.71	0.27	Longirostrine^a^
*Junggarsuchus sloani*	IVPP V14010	(see Ristevski, 2022)	Terrestrial	Solidocrania	0.52	0.54	Brevirostrine
*Macrospondylus bollensis*	BSPG 1984 I258	(see Ristevski, 2022)	Semi‐aquatic	Thalattosuchia	?	0.36	Longirostrine^a^
*Mecistops cataphractus*	TMM M‐3529	(see Ristevski, 2022)	Semi‐aquatic	Crocodylidae	0.72	0.44	Longirostrine
*Metriorhynchus brachyrhynchus*	NHMUK PV OR 32617	(Schwab et al., 2021)	Pelagic	Thalattosuchia	0.4	0.19	Mesorostrine
*Mourasuchus arendsi*	MLP 73‐IV‐15‐9	(Bona et al., 2013)	Semi‐aquatic	Alligatoridae	0.69	0.62	Brevirostrine
*Osteolaemus tetraspis*	FMNH 98386	(see Ristevski, 2022)	Semi‐aquatic	Crocodylidae	0.64	0.39	Mesorostrine
*Plagiophthalmosuchus* cf. *gracilirostris*	NHMUK PV OR 33095	(Schwab et al., 2021)	Pelagic	Thalattosuchia	?	?	Longirostrine^a^
*Protosuchus haughtoni*	BP/1/4770	(see Ristevski, 2022)	Terrestrial	Protosuchidae	0.43	0.49	Brevirostrine
*Rhabdognathus aslerensis*	AMNH FARB 33354	(Erb & Turner, 2021)	Pelagic	Dyrosauria	0.71	0.45	Longirostrine
*Tomistoma dowsoni*	NHMUK PV R 4769	This study	Semi‐aquatic	Gavialoidea	0.72	0.25	Longirostrine
*Tomistoma schlegelii*	TMM M6342	This study	Semi‐aquatic	Tomistominae	0.74	0.22	Longirostrine
*Tomistoma schlegelii*	USNM 211322	(see Ristevski, 2022)	Semi‐aquatic	Tomistominae	0.74	0.22	Longirostrine
*Trilophosuchus rackhami*	QMF 16856	(see Ristevski, 2022)	Terrestrial	Mekosuchinae	0.4	0.66	Brevirostrine

^a^ Inferred.

## 3 RESULTS

### 3.1 Nasal cavity and associated structures

**FIGURE 1:**
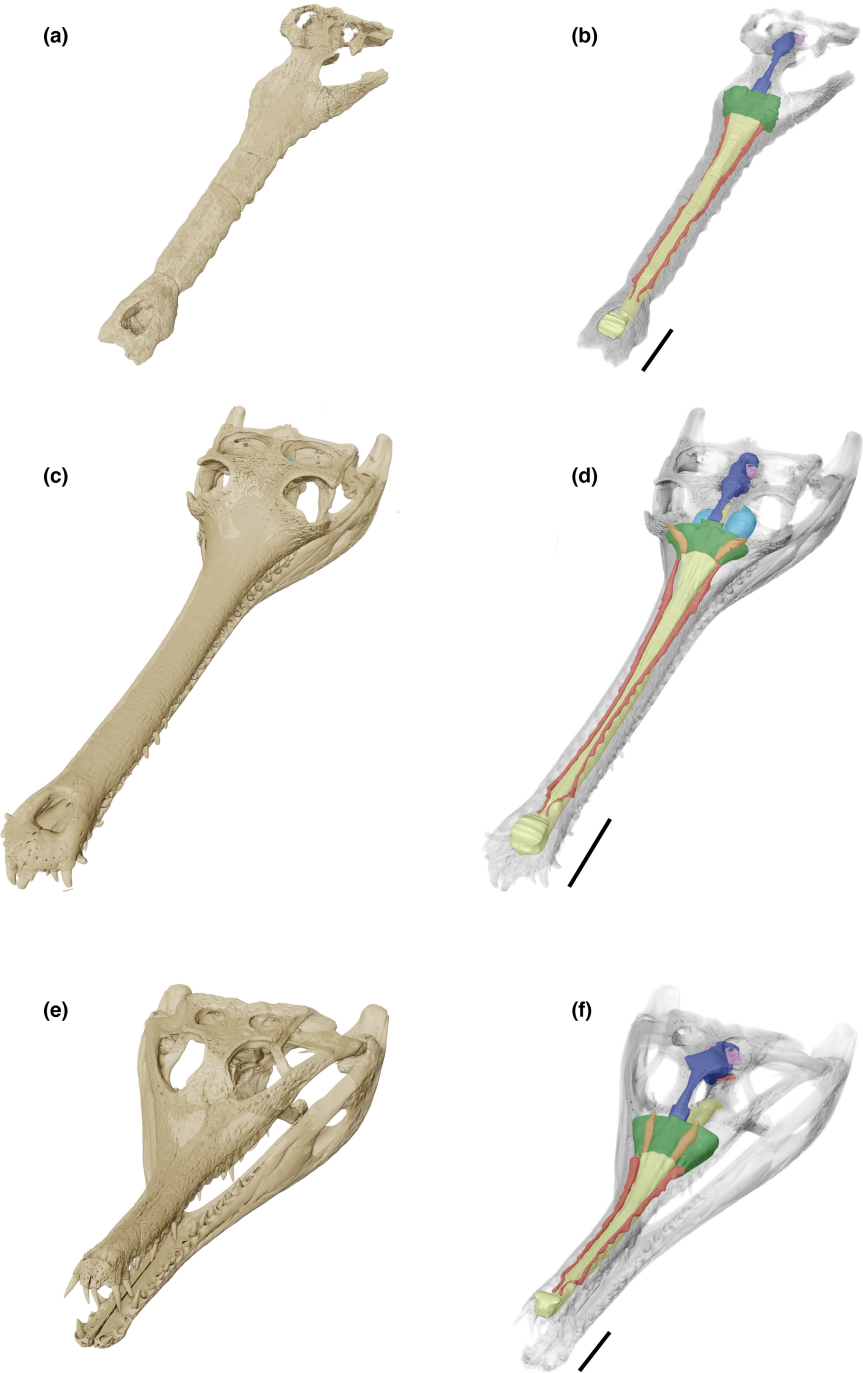
(a) Isosurface rendering of the skull of ‘*Tomistoma*’ *dowsoni* based on NHMUK PV R 4769; (b) reconstruction of the neuroanatomy and neurosensory apparatus of ‘*Tomistoma*’ *dowsoni*; (c) isosurface rendering of the skull of *Gavialis gangeticus* based on FLMNH UF 118998; (d) reconstruction of the neuroanatomy and neurosensory apparatus of *Gavialis gangeticus*; (e) isosurface rendering of the skull of *Tomistoma schlegelii* based on TMM M6342; (f) reconstruction of the neuroanatomy and neurosensory apparatus of *Tomistoma schlegelii*. Scale bars = 50 mm.

**FIGURE 2:**
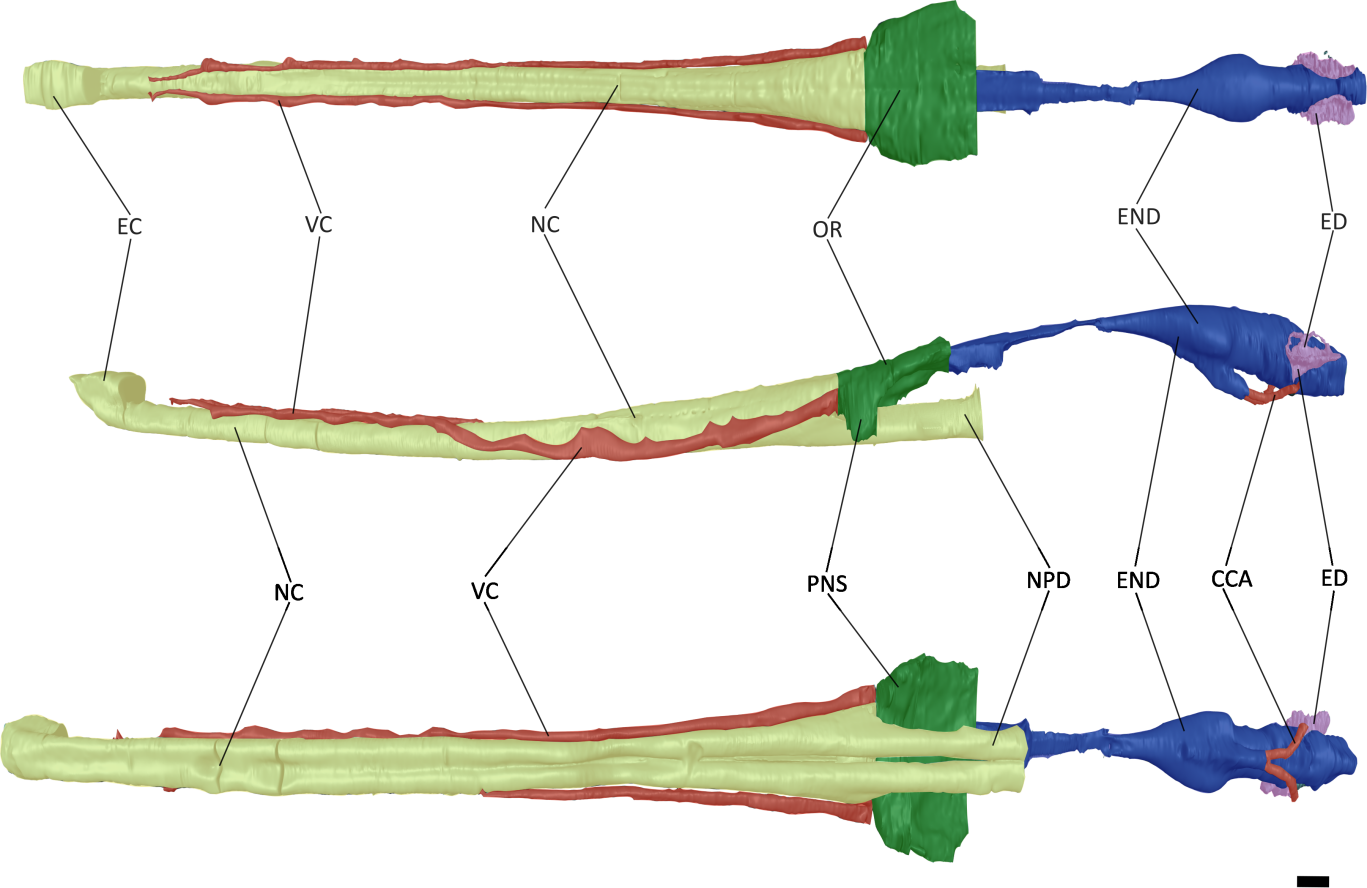
The neuroanatomy of ‘*Tomistoma*’ *dowsoni* (NHMUK PV R 4769) in dorsal, left lateral and ventral views. Abbreviations: CCA, cerebral carotid artery; EC, external choana; ED, endosseous labyrinth; END, encephalic endocast; NC, nasal cavity; NPD, nasopharyngeal duct; OR, olfactory region; PNS, paranasal sinus; VC, neurovascular canal. Scale bar = 10 mm.

**FIGURE 3:**
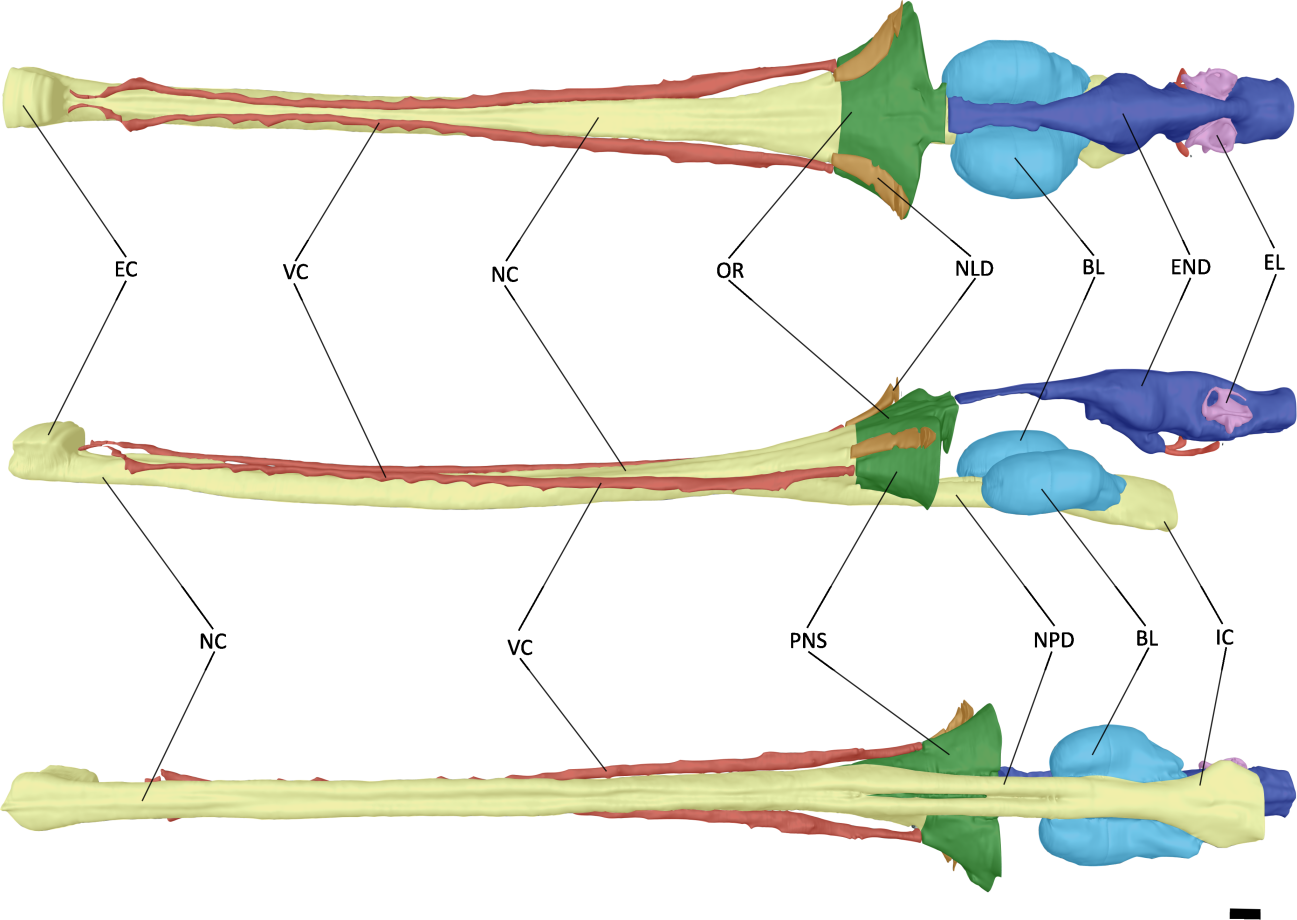
The neuroanatomy of *Gavialis gangeticus* (FLMNH UF 118998) in dorsal, left lateral and ventral views. Abbreviations: BL, pterygoid bulla; CCA, cerebral carotid artery; EC, external choana; ED, endosseous labyrinth; END, endocast; IC, internal choana; NC, nasal cavity; NLD, nasolacrimal duct; NPD, nasopharyngeal duct; OR, olfactory region; PNS, paranasal sinus; VC, neurovascular canal. Scale bar = 10 mm.

**FIGURE 4:**
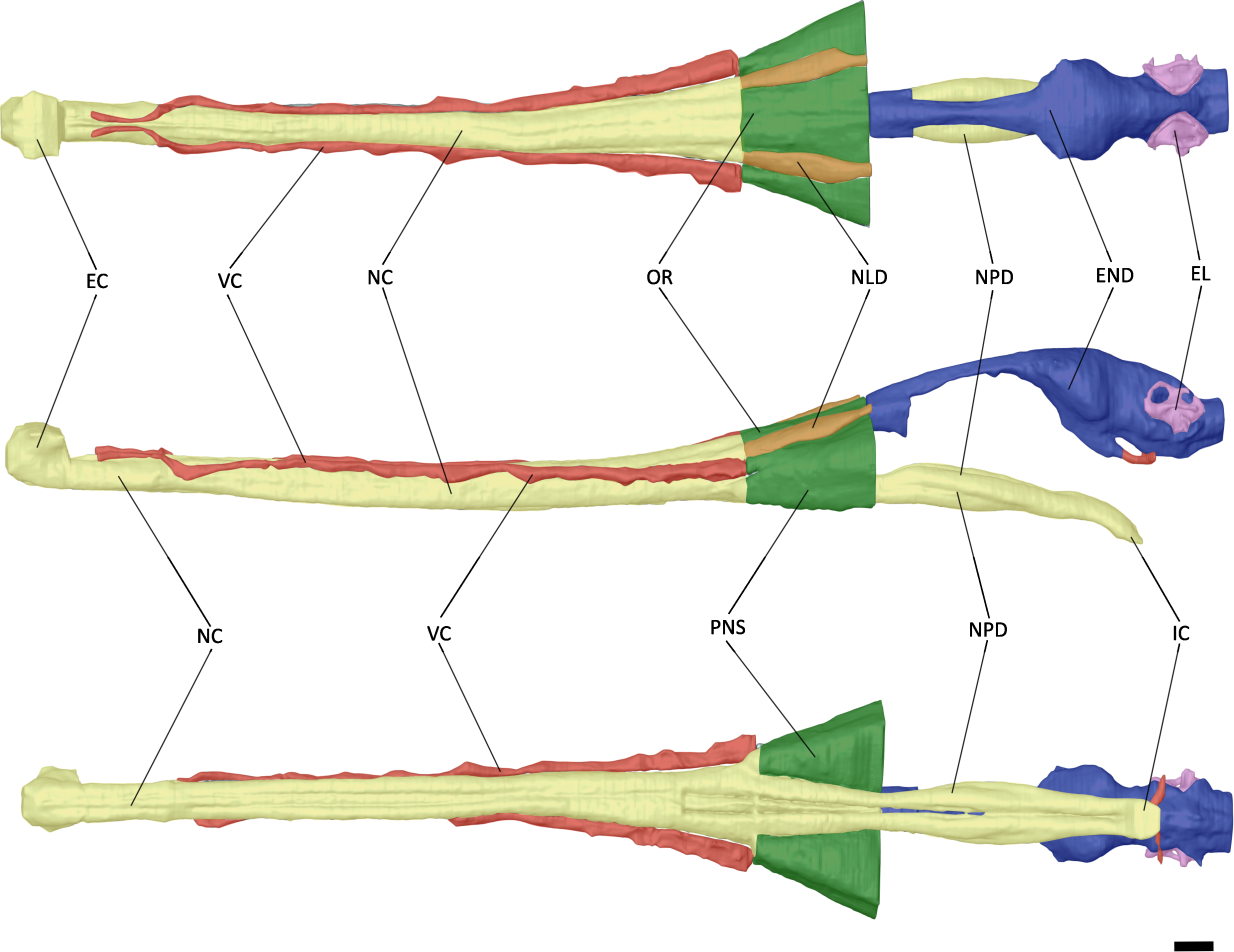
The neuroanatomy of *Tomistoma schlegelii* (TMM M6342) in dorsal, left lateral and ventral views. Abbreviations: CCA, cerebral carotid artery; EC, external choana; ED, endosseous labyrinth; END, endocast; IC, internal choana; NC, nasal cavity; NLD, nasolacrimal duct; NPD, nasopharyngeal duct; OR, olfactory region; PNS, paranasal sinus; VC, neurovascular canal. Scale bar = 10 mm.

The nasal cavity of ‘*Tomistoma’ dowsoni* (NHMUK PV R 4769) generally reflects that of both *Gavialis gangeticus* and *Tomistoma schlegelii*. This morphology is typical of Crocodylomorpha, in that the nasal cavity extends posteriorly from the premaxilla to the basicranium (Pierce et al., 2017; Serrano‐Martínez et al., 2021). At the most anterior part of the snout in NHMUK PV R 4769, *Gavialis gangeticus* and *Tomistoma schlegelii*, the nasal cavity protrudes to form the external naris (Figure 1). Conversely, at its most posterior point, the nasal cavity retracts to form an internal naris in *Gavialis gangeticus* and *Tomistoma schlegelii*; however, this region is too incompletely preserved in NHMUK PV R 4769 to determine whether a similar retraction is present. An extensive, longitudinal midline groove is present along the ventral surface of the nasal cavity in NHMUK PV R 4769, up to the point at which the nasal cavity contacts the paranasal sinus and bifurcates to form the nasopharyngeal duct (Figure 2). The separation of the nasal cavity to form the nasopharyngeal duct also occurs at the point at which the former contacts the paranasal sinus in *Gavialis gangeticus* (Figure 3); however, in *Tomistoma schlegelii*, the nasopharyngeal duct splits posterior to the paranasal sinus, ventral to the encephalic endocast (Figure 4).

The nasal cavity possesses nasal glands in all three species. These glands occupy small concavities located on the ventral surface of the nasals, on the nasomaxillary suture (Cowgill et al., 2022; Witmer, 1995). Whereas they are restricted to the posterior half of the rostrum in *Tomistoma schlegelii* (Cowgill et al., 2022), these concavities extend further anteriorly in both NHMUK PV R 4769 and *Gavialis gangeticus*.

*Gavialis gangeticus* bears a large, egg‐shaped bulla parallel to the nasopharyngeal duct (Figure 3; Martin & Bellairs, 1977; Pierce et al., 2017), with this feature absent in *Tomistoma schlegelii*. As a result of poor preservation of the nasal cavity of NHMUK PV R 4769 ventral to the encephalic endocast, it is unclear whether ‘*Tomistoma*’ *dowsoni* was also characterised by a bulla.

Anterior to the encephalic endocast, the paranasal system expands laterally to form the olfactory region in all three species (Figures 2, 3, 4). This region is rounded and ventrolateral to the nasal passageway, most likely representing the antorbital (=caviconchal) sinus (Fernández & Herrera, 2009; Witmer, 1995). Despite the sinus being closed off in extant crocodylians, this feature is still well developed in those taxa (Pierce et al., 2017). The olfactory bulb connects to the olfactory region in NHMUK PV R 4769, resulting in a sharp contact, as is also the case in *Tomistoma schlegelii*. By contrast, the expansion of the olfactory region is more gradual in *Gavialis gangeticus* (Figure 3). In NHMUK PV R 4769, the olfactory region expands ventrolaterally at the contact between the nasal cavity and the nasopharyngeal duct to form the paranasal sinus (Figure 2). It is unclear whether this morphology is a preservational artefact of NHMUK PV R 4769, given that the entirety of the paranasal sinus protrudes dorsolaterally over the nasopharyngeal duct in *Tomistoma schlegelii* (Figure 4), whereas the paranasal sinus only protrudes dorsolaterally over the nasopharyngeal duct in its most posterior part in *Gavialis gangeticus* (Figure 3). This expansion results in depressions on the internal surface of the prefrontal and lacrimal in the two extant gavialoids, likely due to enlargement of the nasal salt glands (Cowgill et al., 2022; Pierce et al., 2017). In all three species, the dorsal surface of the olfactory region is characterised by a shallow midline groove.

Both extant gharials are characterised by two channel‐like nasolacrimal ducts that extend along the dorsal surface of the olfactory region (Pierce et al., 2017). However, it is unclear if NHMUK PV R 4769 genuinely lacks this feature, or if it is just not preserved. The morphology of the nasolacrimal ducts differs between the two extant gavialoids: in *Gavialis gangeticus* each duct curves posteriorly at the point where the olfactory region expands (Figure 3), whereas they are straight and run parallel to one another in *Tomistoma schlegelii* (Figure 4).

The nasal cavity of all three species possesses two channels that extend from the olfactory region to the most anterior part of the nasal cavity. These channels have been referred to as neurovascular canals, and, more specifically, dorsal alveolar canals or ducts for the trigeminal nerve and maxillary veins and arteries (Pierce et al., 2017; Serrano‐Martínez et al., 2019, 2021). In both extant gharials, these channels run anteroposteriorly on the lateral surfaces of the nasal cavity, with the channels meeting posterior to the external naris, on the dorsal surface of the nasal cavity (Figures 3 and 4). These two channels also run on the lateral surfaces of the nasal cavity in NHMUK PV R 4769; however, they do not meet on the dorsal surface (Figure 2).

The paranasal system of *Gavialis gangeticus*, *Tomistoma schlegelii* and NHMUK PV R 4769 is characterised by a relatively simple morphology, which is representative of longirostrine crocodyliforms (Witmer, 1997; Witmer & Ridgely, 2008). The morphology of the paranasal system of brevirostrine crocodyliforms differs to that of longirostrine crocodyliforms, as the paranasal sinus is broader, the external naris has a greater dorsolateral expansion, the dorsal alveolar ducts have multiple smaller ‘channels’ branching off the main duct, and there is a much larger, pronounced antorbital sinus in the former grouping (Serrano‐Martínez et al., 2021; Witmer, 1997; Witmer & Ridgely, 2008).

### 3.2 Endocranium

**FIGURE 5:**
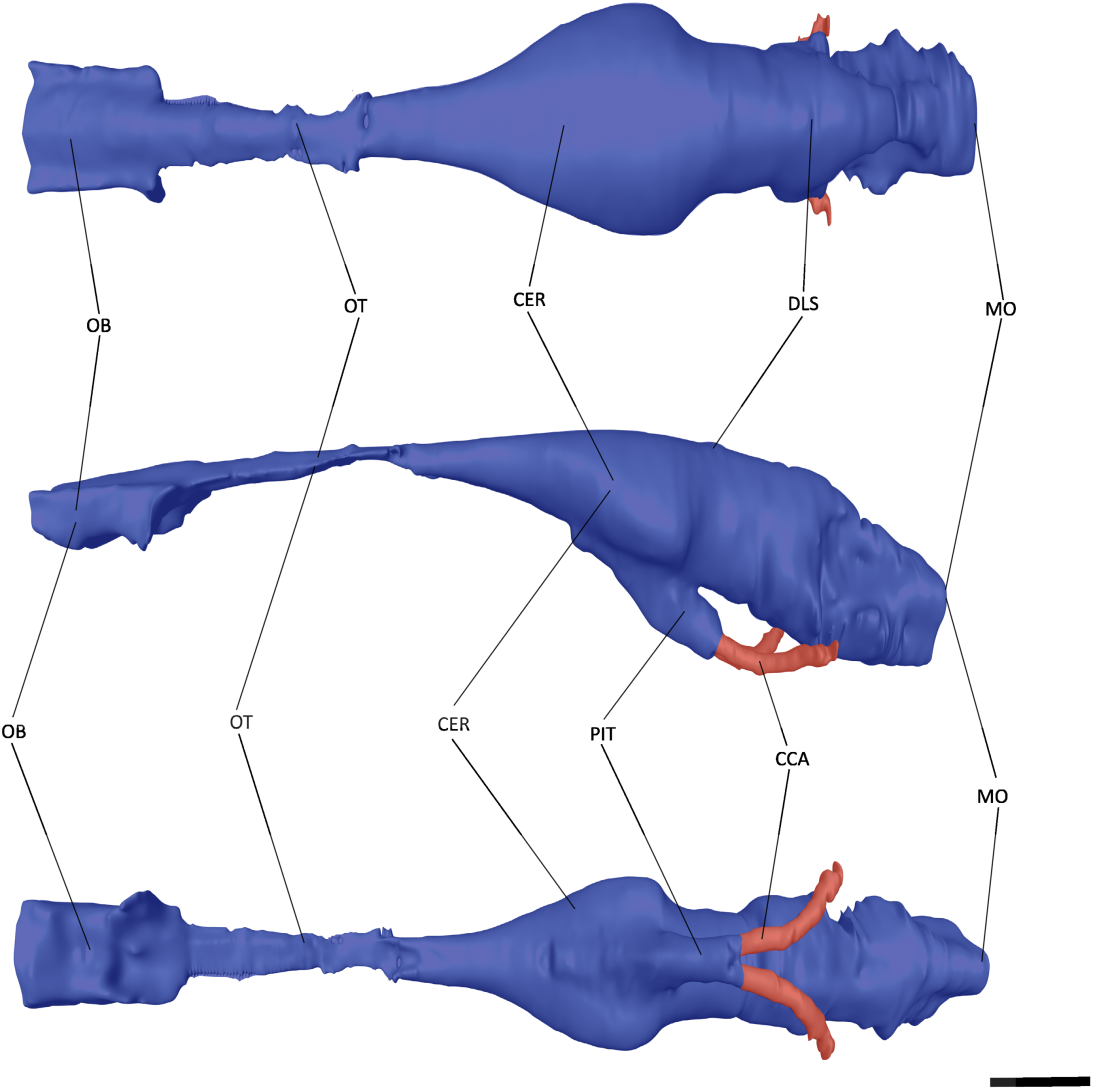
The endocast of ‘*Tomistoma*’ *dowsoni* (NHMUK PV R 4769) in dorsal, left lateral and ventral views. Abbreviations: CCA, cerebral carotid artery; CER, cerebrum; DLS, dorsal longitudinal sinus; MO, medulla oblongata; OB, olfactory bulb; OT, olfactory tract. Scale bar = 10 mm.

**FIGURE 6:**
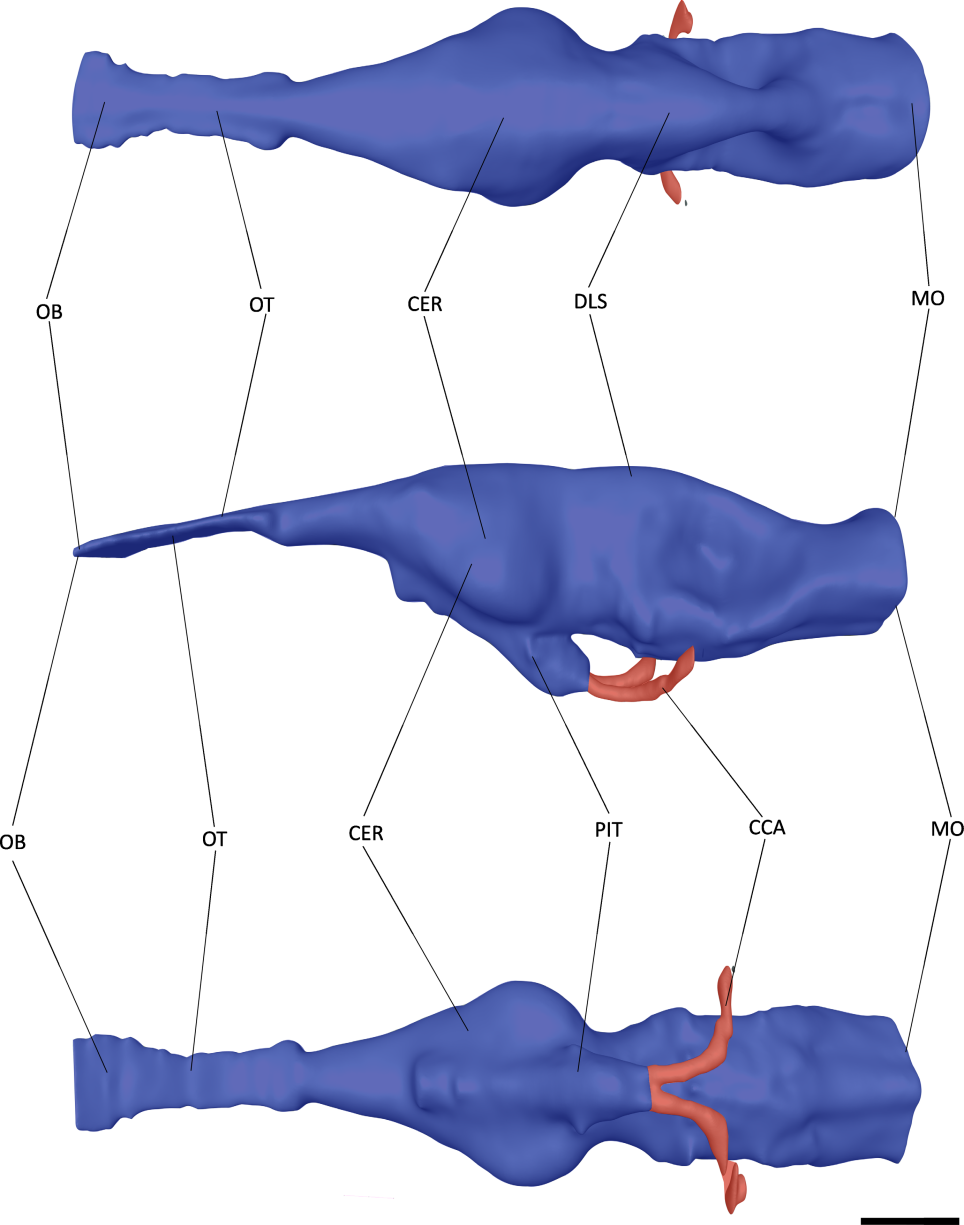
The endocast of *Gavialis gangeticus* (FLMNH UF 118998) in dorsal, left lateral and ventral views. Abbreviations: CCA, cerebral carotid artery; CER, cerebrum; DLS, dorsal longitudinal sinus; MO, medulla oblongata; OB, olfactory bulb; OT, olfactory tract. Scale bar = 10 mm.

**FIGURE 7:**
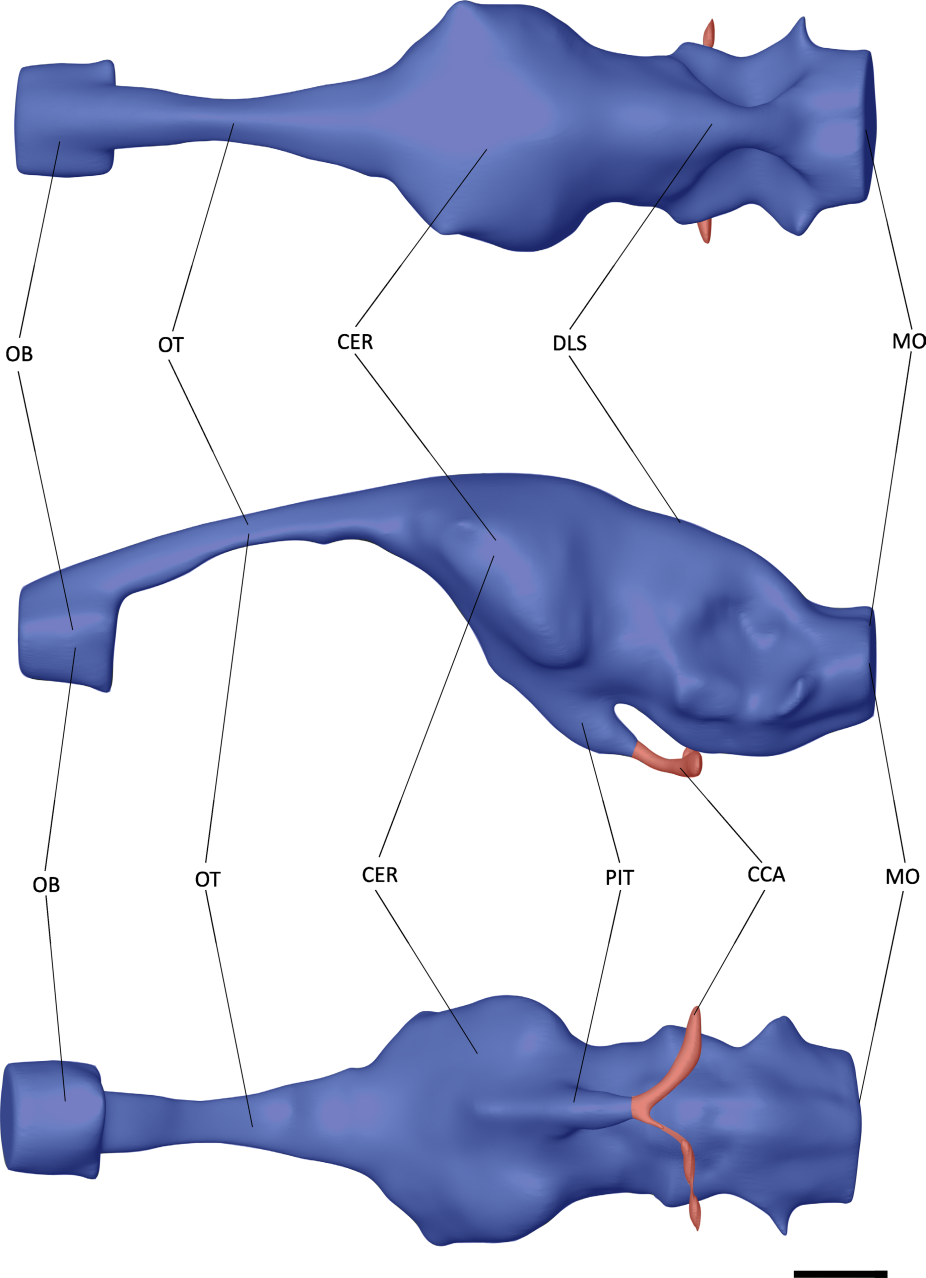
The endocast of *Tomistoma schlegelii* (TMM M6342) in dorsal, left lateral and ventral views. Abbreviations: CCA, cerebral carotid artery; CER, cerebrum; DLS, dorsal longitudinal sinus; MO, medulla oblongata; OB, olfactory bulb; OT, olfactory tract. Scale bar = 10 mm.

Crocodylian encephalic endocasts tend to be relatively straight in outline, with little curvature (Edinger, 1938; Hopson & Gans, 1979), including that of *Gavialis gangeticus*. *Tomistoma schlegelii*, however, appears to be the exception, showing greater curvature, as reflected in acute cephalic and pontine flexure angles (Table 1; Figure 4). The encephalic endocast of NHMUK PV R 4769 also shows a greater degree of curvature and more acute cephalic and pontine flexure angles than other crocodylians (Figures 2 and 3), although this is not to the same extent as that of *Tomistoma schlegelii* (Table 1). As is the case in other eusuchians (Bona et al., 2013, 2015; Serrano‐Martínez et al., 2019, 2021), the encephalic endocast of each of the three species in this study is characterised by a sigmoidal morphology in lateral view (Figures 5, 6, 7). The endocast volume of NHMUK PV R 4769 (23,405 mm^3^) is intermediate between the two extant gavialoids, lower than that of *Gavialis gangeticus* (38,309 mm^3^) but exceeding that of *Tomistoma schlegelii* (19,390 mm^3^).

At the most anterior point of the encephalic endocast, the olfactory bulb is connected to the cerebrum via the olfactory tract (Figures 5, 6, 7). A lack of osteological division between the olfactory tract and bulb has been previously reported in extant crocodylians (Witmer et al., 2008; Witmer & Ridgely, 2008); however, these features can be distinguished from one another in both NHMUK PV R 4769 and *Tomistoma schlegelii* (Serrano‐Martínez et al., 2021), with a slight expansion ventrally and laterally in NHMUK PV R 4769, and a greater expansion ventrally in *Tomistoma schlegelii* (Figures 5 and 7). By contrast, the olfactory bulb and tract in *Gavialis gangeticus* are difficult to distinguish from one another (Pierce et al., 2017), with only a slight lateral expansion anterior to the olfactory region (Figures 3 and 6). The olfactory bulb is anteroposteriorly longer relative to the total encephalic endocast length in NHMUK PV R 4769 than in *Gavialis gangeticus* and *Tomistoma schlegelii*.

The cerebrum of NHMUK PV R 4769 is expansive and bulbous in comparison to the rest of the encephalic endocast (Figure 5). It is intermediate between that of *Gavialis gangeticus* and *Tomistoma schlegelii* in terms of the ratio of the cerebrum width to that of the skull width (Table 2). When the cerebrum width is compared to the encephalic endocast length, NHMUK PV R 4769 has a similar value to that of *Gavialis gangeticus*, with both lower than that of *Tomistoma schlegelii* (Table 2). The cerebrum of *Tomistoma schlegelii* has a near‐symmetrical expansion in dorsal view, whereas the greatest expansion in NHMUK PV R 4769 and *Gavialis gangeticus* occurs at the posterior end of the cerebrum (Figures 5, 6, 7). This posterior expansion has also been noted in other crocodylomorphs (Colbert et al., 1946; Edinger, 1938; Hopson & Gans, 1979; Kley et al., 2010; Pierce et al., 2017). Posteroventral to the cerebrum, and anterior to the optic lobes, is the pituitary (Figures 5, 6, 7). In NHMUK PV R 4769, the pituitary is much more laterally expansive than that of *Gavialis gangeticus* or *Tomistoma schlegelii*; however, it has a similar length to these two species (Table 2). The pituitary has two large channels that extend posterolaterally in all three species (Figures 5, 6, 7). These channels curve dorsolaterally at the posterior part in all three species and house the cerebral carotid artery (Dufeau & Witmer, 2015; Hopson & Gans, 1979; Pierce et al., 2017; Witmer et al., 2008).

The optic lobes of the encephalic endocast are difficult to segment in crocodile‐line archosaurs as a result of the thick dural envelope (Hopson & Gans, 1979; Pierce et al., 2017), but they can be deduced from the mesencephalon region of the brain (Serrano‐Martínez et al., 2021). These lobes are more prominent in early ontogenetic stages, becoming less distinct as individuals mature (Hu et al., 2021; Jirak & Janacek, 2017; Ristevski, 2022). Similarly, segmentation of the cranial nerves is difficult in fossil taxa due to both preservation and quality of the scan (Pierce et al., 2017). This is particularly evident in NHMUK PV R 4769 due to the poor preservation in this area (Figure 1a). As a result, this region, as well as the paratympanic sinuses, were not segmented in *Tomistoma schlegelii* and *Gavialis gangeticus*, as there would not have been a direct comparison with NHMUK PV R 4769.

### 3.3 Endosseous labyrinth

**FIGURE 8:**
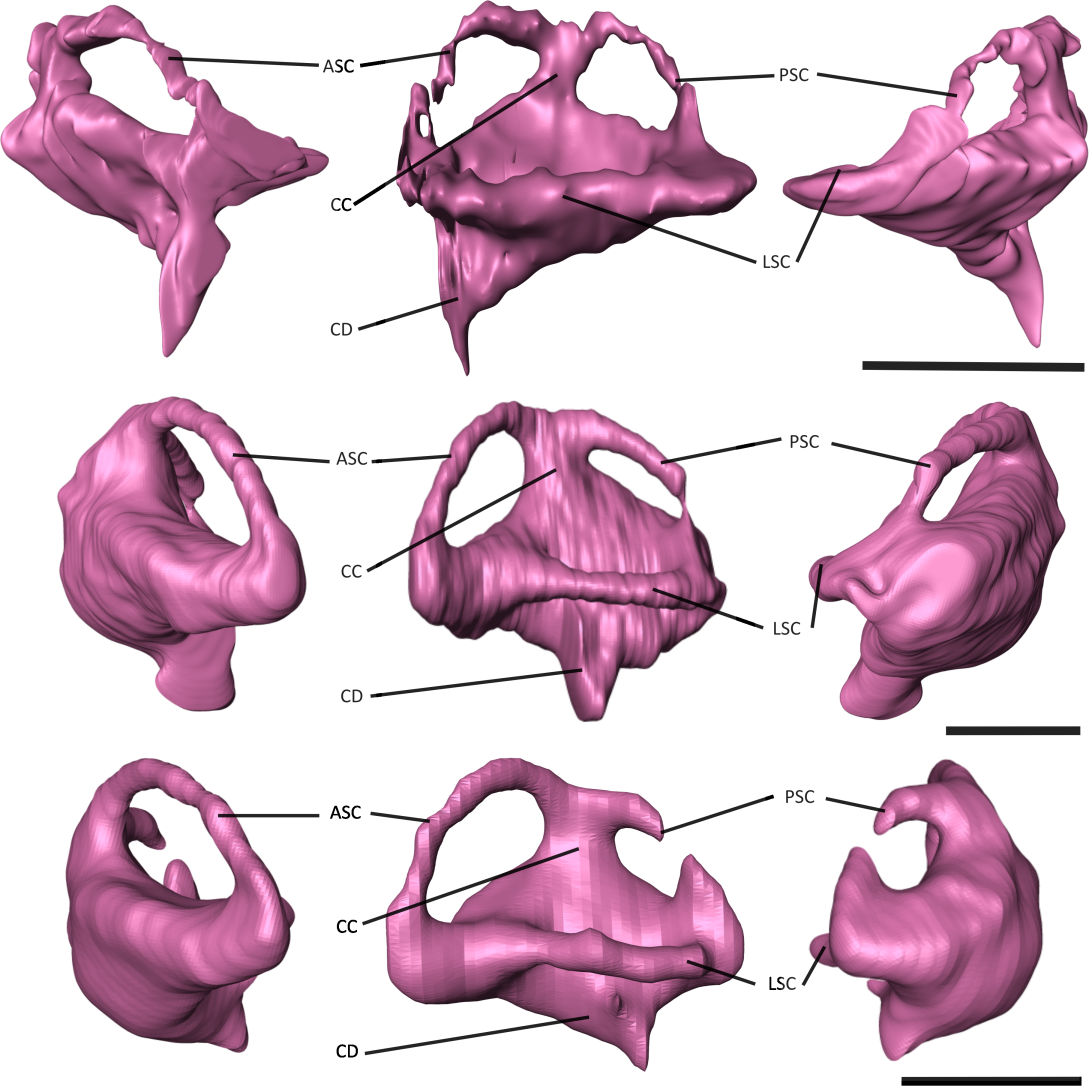
Endosseous labyrinths of A) ‘*Tomistoma*’ *dowsoni* (NHMUK PV R 4769), *Gavialis* gangeticus (FLMNH UF 118998), and *Tomistoma schlegelii* (TMM M6342) in anterior, dorsal and posterior views. Abbreviations: ASC, anterior semi‐circular canal; CC, common crus; CD, cochlear duct; LSC, lateral semi‐circular canal; PSC, posterior semi‐circular canal. Scale bar = 10 mm.

Poor preservation of the region of the skull of NHMUK PV R 4769 in which the endosseous labyrinth would have been housed made segmentation difficult; however, the overall shape could be reconstructed (Figure 8). The endosseous labyrinths of *Gavialis gangeticus* and *Tomistoma schlegelii* have a similar morphology to one another, with the anterior semi‐circular canal being larger than the posterior semi‐circular canal, as is the case in most archosaurs (Brusatte et al., 2016; Witmer et al., 2003), including other extant and extinct crocodylians (Bona et al., 2013, 2015; Dufeau & Witmer, 2015; Georgi & Sipla, 2008; Witmer et al., 2008; Witmer & Ridgely, 2008). In dorsal view, the anterior and posterior semi‐circular canals appear more equidimensional in NHMUK PV R 4769 (Figure 8); however, when the area of each canal is quantified, the anterior semi‐circular canal is more than double that of its posterior counterpart (Table 2), resulting in a similar value to that of *Gavialis gangeticus* (Pierce et al., 2017). The cochlear duct, responsible for auditory capabilities in the inner ear, is of comparable size across the three species in this study (Tables 1 and 2); however, whereas the cochlear duct extends posteroventrally in both the extant gharials, it projects anteroventrally in NHMUK PV R 4769. As a result of the poor preservation of NHMUK PV R 4769 in this region, it is not possible to distinguish the separation of the lateral semi‐circular canal from the common crus.

### 3.4 Ecological capabilities

NHMUK PV R 4769 is estimated to have an olfactory ratio of 1.78, which is higher than that of *Gavialis gangeticus* (1.69), but lower than that of *Tomistoma schlegelii* (1.82). A comparable range of olfactory ratios is seen in other extant crocodylians including *Alligator mississippiensis* (1.76), *Caiman crocodilus* (1.75) and *Crocodylus niloticus* (1.86) (Table 4; Serrano‐Martínez et al., 2021).

**TABLE 4:** Estimation of olfactory acuity in crocodyliforms.

	*Tomistoma dowsoni*	*Tomistoma schlegelii*	*Gavialis gangeticus*	*Agaresuchus fontisensis*	*Alligator mississippiensis*	*Caiman crocodilus*	*Crocodylus niloticus*	*Lohuecosuchus megadontos*	*Osteolaemus tetraspis*
	This study	This study	This study	Serrano‐Martínez et al. (2021)	Serrano‐Martínez et al. (2021)	Serrano‐Martínez et al. (2021)	Serrano‐Martínez et al. (2021)	Serrano‐Martínez et al. (2021)	Serrano‐Martínez et al. (2021)
Olfactory bulb (mm)	17.59	19.13	16.65	17.81	17.30	10.92	20.37	20.80	15.43
Cerebral hemisphere (mm)	29.19	28.89	33.29	30.34	29.80	19.62	30.50	33.52	24.90
Olfactory ratio	**1.78**	**1.82**	**1.69**	**1.88**	**1.76**	**1.75**	**1.82**	**1.79**	**1.79**

The relative size of the optic region, estimated as the ratio of the volume of the optic lobes to that of the encephalic endocast, averages between 10% and 15% in most extant crocodylians, with that of *Tomistoma schlegelii* and NHMUK PV R 4769 estimated to be 11% and 12% respectively (Table 5). By contrast, the relative volume for *Gavialis gangeticus* is estimated to be 18% (Table 5).

**TABLE 5:** Estimation of the relative size of the optic region of crocodyliforms.

	*Tomistoma dowsoni*	*Tomistoma schlegelii*	*Gavialis gangeticus*	*Agaresuchus fontisensis*	*Alligator mississippiensis*	*Caiman crocodilus*	*Crocodylus niloticus*	*Lohuecosuchus megadontos*	*Osteolaemus tetraspis*
	This study	This study	This study	Serrano‐Martínez et al. (2021)	Serrano‐Martínez et al. (2021)	Serrano‐Martínez et al. (2021)	Serrano‐Martínez et al. (2021)	Serrano‐Martínez et al. (2021)	Serrano‐Martínez et al. (2021)
Endocast volume (mm^3^)	23,405.1	19,390.5	38,309.9	14,696.4	18,719.9	4941.7	24,764.8	13,604.5	11,593.1
Optic lobe volume (mm^3^)	2868.1	2105.7	6996.5	2263.6	2467.9	936.5	3789.0	1885.9	2577.4
Relative volume (%)	**12**	**11**	**18**	**15**	**13**	**19**	**15**	**15**	**22**

The REQ of NHMUK PV R 4769 is estimated as 0.86, lower than that of *Gavialis gangeticus* (1.22) and *Tomistoma schlegelii* (1.04) (Table 6). However, Wharton's (Wharton, 2002) study on *Gavialis gangeticus* showed that the REQ ranges from 0.8 to 2.0 in this species, and the REQ of euschian species sampled by Serrano‐Martínez et al. (2021) ranges from 0.9 to 1.2 (Table 6).

**TABLE 6:** Estimation for the reptile encephalisation quotient (REQ) for crocodyliforms.

	*Tomistoma dowsoni*	*Tomistoma schlegelii*	*Gavialis gangeticus*	*Agaresuchus fontisensis*	*Alligator mississippiensis*	*Caiman crocodilus*	*Crocodylus niloticus*	*Lohuecosuchus megadontos*	*Osteolaemus tetraspis*
	This study	This study	This study	Serrano‐Martínez et al. (2021)	Serrano‐Martínez et al. (2021)	Serrano‐Martínez et al. (2021)	Serrano‐Martínez et al. (2021)	Serrano‐Martínez et al. (2021)	Serrano‐Martínez et al. (2021)
Endocast volume (cm^3^)	23.41	19.39	38.31	14.70	18.72	4.94	24.76	13.60	11.59
Brain volume (cm^3^)	8.19	7.19	11.69	5.90	7.01	3.71	8.56	5.58	4.98
Body mass (g)	168,653.9	93,619.6	170,000.0	58,107.02	104,328.28	5887.67	151,389.30	88,719.46	20,228.39
REQ	**0.86**	**1.04**	**1.22**	**0.91**	**0.96**	**1.71**	**0.96**	**0.83**	**1.63**

### 3.5 Landmark‐based morphometrics

**FIGURE 9:**
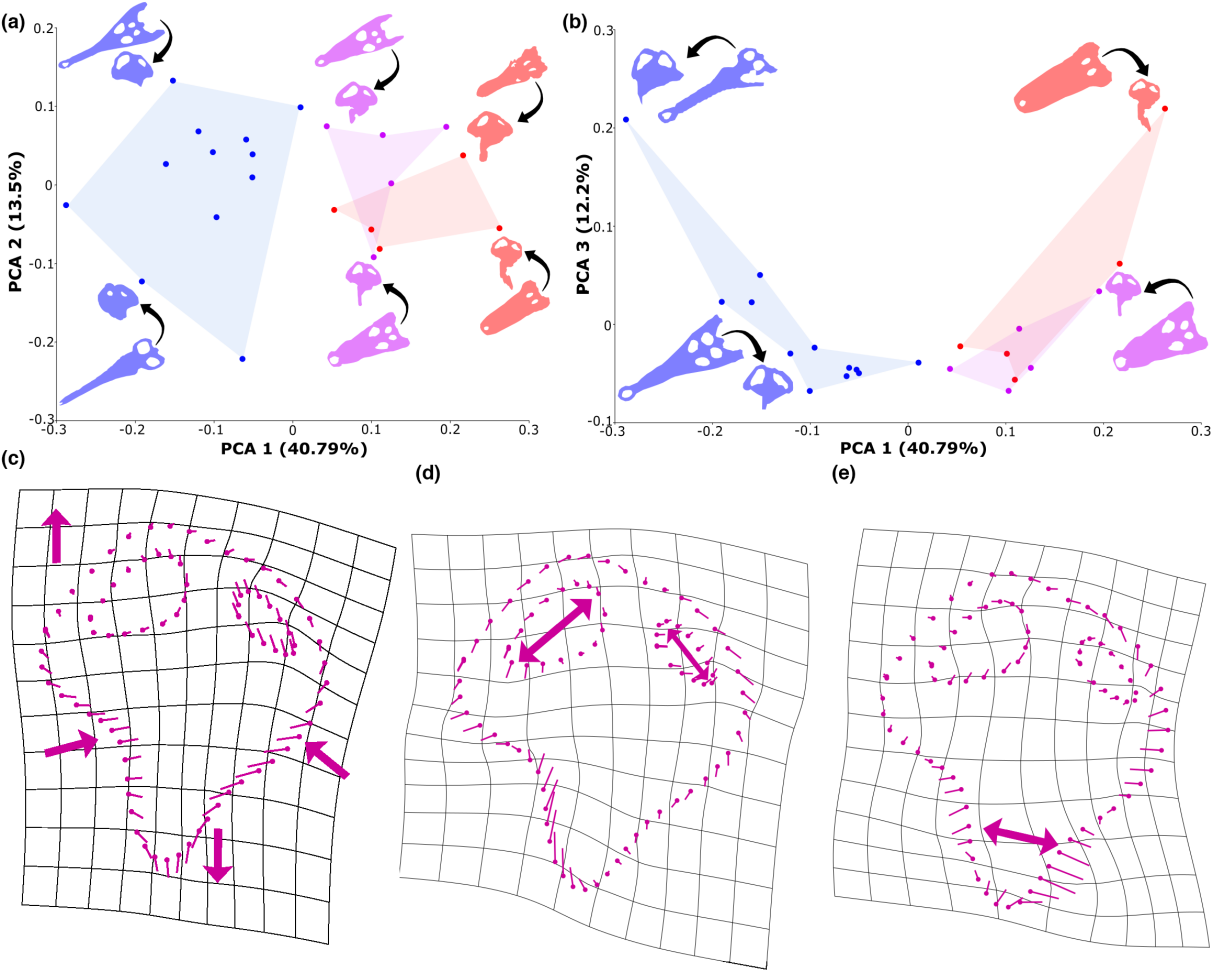
Principal component analysis (PCA) showing the variation in the shape of the endosseous labyrinth in 20 species of Crocodylomorpha. (a) shows the variation between PC1 versus PC2, (b) shows the variation between PC1 versus PC3. Skull morphology is highlighted in blue for longirostrine taxa, pink for mesorostrine taxa and red for brevirostrine taxa (see Table 3). (c) shows the shape variation in PC1, d) variation in PC2 and (e) variation in PC3. Arrows indicate the direction of change in the endosseous labyrinth. Skull silhouettes in (a) *Tomistoma schlegelii* (TMM M6342) and *Cricosaurus araucanensis* (MLP 72‐IV‐7‐1) (blue); *Crocodylus rhombifer* (NMB AB50.0171) and *Osteolaemus tetraspis* (FMNH 98386) (pink); *Junggarsuchus sloani* (IVPP V14010) and *Mourasuchus arendsi* (MLP 73‐IV‐15‐9) (red); (b) ‘*Tomistoma*’ *dowsoni* (NHMUK PV R 4769) and *Gunggamarandu maunala* (QMF 548) (blue); *Crocodylus rhombifer* (NMB AB50.0171) (pink); *Mourasuchus arendsi* (MLP 73‐IV‐15‐9) (red).

The endosseous labyrinth varies mostly in its width and height across crocodyliforms. The first three principal components (PCs) equate to approximately 66.5% of endosseous labyrinth shape variation, with only the first five principal components explaining more than 5% of variation (Figure 9). PC1 characterises approximately 40.8% of variation, with positive values indicating dorsoventrally high and anteroposteriorly short labyrinths, which characterises taxa such as *Mourasuchus arendsi* and *Junggarsuchus sloani* (Figure 9c). Conversely, negative values indicate dorsoventrally low and anteroposteriorly long labyrinths, which characterises taxa such as ‘*Tomistoma*’ *dowsoni* and *Tomistoma schlegelii* (see Figure 8). PC2 characterises 13.6% of variation, with positive values indicating a greater difference in the size of the anterior semi‐circular canal to its posterior counterpart, as seen in *Tomistoma schlegelii* and *Osteolaemus tetraspis* (Figure 9d), with positive values indicating equidimensional canals, such as in the thalattosuchians *Cricosaurus araucanensis* and *Plagiophthalmosuchus* cf. *gracilirostris*. PC3 characterises 12.2% of variation, with positive values indicating wider, anterior‐directed cochlear ducts, such as in ‘*Tomistoma*’ *dowsoni* and *Mourasuchus arendsi*, and negative values indicating narrower, ventrally directed cochlear ducts, such as in *Mecistops cataphractus* and *Gunggamarandu maunala* (Figure 9e).

When broadly classified into their environmental habitats, there is a large overlap in semi‐aquatic, terrestrial and pelagic taxa (Table 3), although this is most likely because of the higher number of the sampled species assigned to a semi‐aquatic ecology in this study. When classified based on their skull shape (Table 3), distinct clusters of labyrinth shapes can be observed, with crocodyliforms categorised as longirostrine forming one grouping, and taxa with brevirostrine and intermediate skull morphologies forming a second grouping (Figure 9a,b).

## 4 DISCUSSION

### 4.1 Ecological versus phylogenetic signal

Features of the neuroanatomy and neurosensory apparatus of gavialoids appear to show an ecological and/or phylogenetic signal. Below we discuss several of these features, including their potential implications for reconstructing the phylogenetic relationships and macroecology of gavialoids and other crocodyliforms.

The thickness of the semi‐circular canals of the endosseous labyrinths for example, is thought to be dependent on the ecology of crocodylomorph species (Schwab et al., 2020). Whereas terrestrial species have a dorsoventrally tall labyrinth, with thin semi‐circular canals, and pelagic species have a compact labyrinth, semi‐aquatic crocodyliforms have an intermediate labyrinth morphology (Schwab et al., 2020), which characterises ‘*Tomistoma*’ *dowsoni*, *Gavialis gangeticus* and *Tomistoma schlegelii* (Figure 8). The endosseous labyrinth appears to be anteroposteriorly wider and dorsoventrally shorter in longirostrine crocodyliforms, but narrower and taller in brevirostrine taxa (Figure 9b). More species, particularly fossil crocodylomorphs, need to be included in analyses to test this more robustly; however, this preliminary finding potentially indicates that snout and skull morphology, which are themselves partly constrained by ecology in crocodylomorphs, exert an influence on the shape of the endosseous labyrinth in this group. Recent studies have also recognised the influence of skull shape on the endosseous labyrinth in turtles (Evers et al., 2022) and on braincase shape in birds (Chiappe et al., 2022), suggesting that this might be a more widespread pattern.

The relatively large size of the cerebrum in birds and mammals has been associated with refined sensory inputs in these groups, as a larger cerebral region implies a greater neuronal area to execute complex behaviours (Pierce et al., 2017; Rogers, 1999). When comparing cerebrum width to skull width, *Tomistoma schlegelii* has a higher value than ‘*Tomistoma*’ *dowsoni* and *Gavialis gangeticus* (Table 1), which could suggest that *Tomistoma schlegelii* has greater behavioural complexity, as also reflected in the higher olfactory acuity estimation for this species (Table 4). Ecological studies have suggested that *Tomistoma schlegelii* shows complex behavioural patterns during courtship, for example, employing visual, tactile and auditory cues (Staniewicz et al., 2022). Analysis of the vocalisations produced by the two living gavialoids has revealed differences between their call structures (Bonke et al., 2015), with sounds produced by *Tomistoma schlegelii* having different acoustic properties and context, with a greater reliance on visual or olfactory cues, particularly in underwater environments (Staniewicz et al., 2022). These differences in underwater signals have been suggested to result from morphological differences (Dinets, 2013; Staniewicz et al., 2022), with a possible role for the larger cerebrum in *Tomistoma schlegelii*. *Gavialis gangeticus* and ‘*Tomistoma*’ *dowsoni* share a similar cerebrum morphology, in which its greatest expansion occurs posteriorly, whereas that of *Tomistoma schlegelii* has a symmetrical expansion (Figures 5, 6, 7). Although both extant gharials occupy aquatic habitats, *Gavialis gangeticus* is observed in streams and rivers with sandy, grassy or rocky shores, whereas *Tomistoma schlegelii* is observed in densely vegetated swamps and lowland forest rivers (Staniewicz et al., 2022). The palaeoenvironment of the Moghra Formation, which ‘*Tomistoma*’ *dowsoni* inhabited, is thought to be a tide‐dominated estuary (Georgalis et al., 2020), closer to the environments inhabited by *Gavialis gangeticus*. As such, it is possible that cerebrum morphology might correspond to differences in ecology. Extant species such as *Alligator mississippiensis*, *Crocodylus niloticus* and *Crocodylus johnstoni* are characterised by a cerebrum morphology that is similar to *Gavialis gangeticus* and ‘*Tomistoma*’ *dowsoni* (see Serrano‐Martínez et al., 2021; Witmer et al., 2008), whereas extinct species such as *Pelagosaurus typus* and *Rhabdognathus aslerensis* share a closer cerebrum morphology with *Tomistoma schlegelii* (see Erb & Turner, 2021; Pierce et al., 2017), perhaps indicating a phylogenetic signal that has been overprinted by ecology. However, evaluations of more fossil crocodylomorphs will be necessary to more robustly test this hypothesis.

The nasal cavity endocast of ‘*Tomistoma*’ *dowsoni* generally reflects those of longirostrine crocodyliforms, specifically the two extant gavialoids. These are characterised by a relatively simple morphology, comprising a long and narrow nasal cavity which bifurcates, forming the nasopharyngeal duct anteriorly, an expanded paranasal sinus, and two dorsal alveolar ducts. Brevirostrine crocodyliforms, on the other hand, have more complex apparatuses (see Serrano‐Martínez et al., 2021; Witmer, 1997; Witmer & Ridgely, 2008). In both *Gavialis gangeticus* and *Tomistoma schlegelii*, the nasolacrimal ducts are located on the dorsal surface of the olfactory region, which is not seen in early marine longirostrine taxa (Pierce et al., 2017), however, their morphology differs. Similarly, the external naris, which has a dorsal inflection, is not seen in early longirostrine crocodylomorphs, but characterises eusuchians (Pierce et al., 2017; Serrano‐Martínez et al., 2021). The morphology of this dorsal inflection also differs between longirostrine and brevirostrine eusuchians (Figures 2, 3, 4; see Serrano‐Martínez et al., 2021). Additionally, and by contrast with brevirostrine crocodylians (Serrano‐Martínez et al., 2021), ‘*Tomistoma*’ *dowsoni*, *Gavialis gangeticus* and *Tomistoma schlegelii* lack a large antorbital sinus, nor are there smaller channels branching off the dorsal alveolar canals. Evaluation of the nasal endocast in more fossil gavialoids is therefore required, given that the morphology of all of these features could reflect an ecological and/or phylogenetic signal in this group.

Evaluation of additional gavialoid species, such as *Hanyusuchus sinensis* (Iijima et al., 2022), will also be crucial in determining whether the ‘intermediate’ morphology seen in ‘*Tomistoma*’ *dowsoni* is also reflected in these taxa. Furthermore, there is evidence for a pterygoid bulla in *Hanyusuchus sinensis* (Iijima et al., 2022), a feature that characterises *Gavialis gangeticus* (Martin & Bellairs, 1977), but not *Tomistoma schlegelii*, and that has also been identified in extinct species of *Gavialis* (*Gavialis lewisi* and *Gavialis bengawanicus*), as well as the extinct gavialoid *Eogavialis africanum* from the late Eocene of Egypt (Hecht & Malone, 1972; Iijima et al., 2022; Lull, 1944; Martin et al., 2012). It might also be present in several South American gryposuchine gavialoids, including *Dadagavialis gunai* and *Gryposuchus* (Riff & Aguilera, 2008; Salas‐Gismondi et al., 2016, 2019). In Rio and Mannion's (2021) phylogenetic analysis, ‘*Tomistoma*’ *dowsoni* is recovered as the sister taxon to a clade that includes *Eogavialis africanum*, *Gavialis* and gryposuchines. Given that *Eogavialis africanum* is potentially a ‘problematic’ taxon in terms of its temporal incongruence with the reconstructed divergence date of *Gavialis* and *Tomistoma*, it will therefore be informative to determine if a bulla is truly synapomorphic of this clade, or is more widespread among gavialoids, with either more than one independent origin of the bulla, or its apomorphic loss in *Tomistoma schlegelii*.

### 4.2 Systematics of ‘*Tomistoma*’ *dowsoni* and contemporaneous gavialoids

Coupled with the results from recent phylogenetic analyses (Groh et al., 2020; Rio & Mannion, 2021), the neuroanatomy of ‘*Tomistoma*’ *dowsoni* further suggests this species is more closely related to *Gavialis gangeticus* than to *Tomistoma schlegelii*, and thus casts additional doubt as to its current generic attribution. Similarly, revision of contemporaneous Miocene species from the Mediterranean region that have previously been referred to *Tomistoma* indicates that none of them share close affinities with the extant species either (Nicholl et al., 2020). An anatomical and taxonomic revision of ‘*Tomistoma*’ *dowsoni*, including the type material (Fourtau, 1920), is currently in preparation, along with ongoing systematic work on the Miocene gavialoids of Europe and North Africa (Burke et al., 2022).

## 5 CONCLUSIONS

Our reconstruction of the neuroanatomy of the Miocene North African gavialoid ‘*Tomistoma*’ *dowsoni* demonstrates that it displays an intermediate morphology between the two extant gavialoids, *Gavialis gangeticus* and *Tomistoma schlegelii*. This morphology is relatively simple, with similar shaped endocasts seen in all three species. Features such as the endosseous labyrinth and the cerebrum appear to have morphologies that are primarily influenced by ecology. By contract, the presence of a pterygoid bulla in *Gavialis* and other closely related gavialoids, but its absence in *Tomistoma*, could potentially reflect a phylogenetic signal of crocodylians more closely related to *Gavialis* than to *Tomistoma*. Comparison of the neuroanatomy and neurosensory apparatus of ‘*Tomistoma*’ *dowsoni* to the two extant gavialoids has potentially revealed more features that could be interpreted as an ecological or phylogenetic signal; however, the evaluation of more fossil gavialoids is needed to more robustly test such hypotheses. Finally, our study supports the placement of ‘*Tomistoma*’ *dowsoni* as phylogenetically closer to *Gavialis gangeticus* than to *Tomistoma schlegelii*, highlighting the need for a taxonomic revision of this fossil species.

## AUTHOR CONTRIBUTIONS

PMJB segmented NHMUK PV R 4769, analysed the data, produced the figures and wrote the manuscript. PDM conceived the project idea and contributed to writing and revising the manuscript.

## FUNDING INFORMATION

PDM's contribution was supported by grants from The Royal Society (UF160216 and RGF\EA\201037) and The Leverhulme Trust (RPG‐2021‐2022).

## Data Availability

Data will be made openly available in a public repository after article publication.
